# Repetitive stress fracture: a warning sign of genetic susceptibility to fracture? A case report of a heterozygous variant in *SERPINF1*

**DOI:** 10.20945/2359-3997000000375

**Published:** 2021-07-16

**Authors:** Mariana Lima Mascarenhas Moreira, Iana Mizumukai de Araújo, Greice Andreotti de Molfetta, Wilson Araújo Silva, Francisco José Albuquerque de Paula

**Affiliations:** 1 Universidade de São Paulo Faculdade de Medicina de Ribeirão Preto Departamento de Clínica Médica Ribeirão Preto SP Brasil Departamento de Clínica Médica, Faculdade de Medicina de Ribeirão Preto, Universidade de São Paulo, Ribeirão Preto, SP, Brasil.; 2 Universidade de São Paulo Faculdade de Medicina de Ribeirão Preto Departamento de Genética Ribeirão Preto SP Brasil Departamento de Genética, Faculdade de Medicina de Ribeirão Preto, Universidade de São Paulo, Ribeirão Preto, SP, Brasil.

## Abstract

The occurrence of fractures in young individuals is frequently overlooked by physicians, especially when associated with exercise or trauma. Nevertheless, multiple fractures should always be investigated since underlying conditions can predispose to such events. We describe here the case of a young, healthy woman who sustained multiple fractures in the lower limbs, which were initially considered to be “stress fractures”. Further investigation, including a panel of genes associated with osteogenesis imperfecta, revealed that the patient is a heterozygous carrier of a *SERPINF1* variant. According to criteria recommended by the American College of Medical Genetics and Genomics and the Association for Molecular Pathology, this variant is classified as likely benign (PM2, PP3, PP4, BP1, and BP4). The patient's mother and brother were also asymptomatic carriers of the variant and had sustained previous minor fractures. The patient had normal biochemical profile and bone density. This condition has been rarely described and is not associated with low bone mineral density or altered bone turnover markers. This case highlights the importance of investigating multiple fractures in young patients who are otherwise healthy since these may be a warning sign of rare genetic conditions associated with fragility fractures.

## INTRODUCTION

The term “stress fracture” encompasses two distinct processes. One is due to excessive and repetitive load on the skeleton, known as “fatigue fracture”, which was first described almost two centuries ago in Prussian soldiers ( 1 ). The other one occurs under normal load in weak bone and is known as “insufficiency fracture”. Fatigue fractures occur typically in young subjects, most frequently women, and in approximately 75% of the cases affect the tibia, tarsal bones, and metatarsals. We report here the case of a young woman who sustained, in conditions of moderate exercise, four fractures with mixed components of fatigue and insufficiency fractures.

Genetics is a major determinant of bone strength and influences both bone mass and microstructural properties. Similar to other complex diseases, primary osteoporosis has a genetic background associated with multiple genetic variants across several genes, each having small contributions to bone phenotype. On the other hand, rare genetic diseases, in which variants in a single gene can cause severe bone fragility, offer a window of opportunity for unveiling molecular mechanisms directly involved in fracture susceptibility and development of new therapeutic drugs for osteoporosis. Repeated episodes of fragility fractures in young individuals are natural signs for researchers and clinicians pursuing advances in the science of the mechanisms, diagnosis, and therapy of osteoporosis. The last decades have led to the identification of several genetic defects driving bone fragility disorders. For example, variants in *LRP5* ( 2 ) and *WNT1* ( 3 ), which in the homozygous state cause the osteoporosis-pseudoglioma syndrome and a severe form of osteogenesis imperfecta (OI), respectively, are associated with early-onset osteoporosis in heterozygous conditions. The recessive homozygous variants in the *SERPINF1* gene were originally described as the cause of type VI OI, a moderate to severe disease with a progressive course and poor response to bisphosphonates. However, previous reports have shown heterozygous carriers of the *SERPINF1* variant without abnormal areal bone density, volumetric bone density, or markers of bone metabolism ( 4 ), suggesting the absence of skeletal disorders, although data on fractures was not presented.

We report here a case of a phenotypically healthy young woman whose clinical manifestations – four sequential fractures elicited by mild or at the most moderate exercise – combined components of fatigue and insufficiency fractures. The present study points out the mild impairment in bone strength among individuals exhibiting heterozygous *SERPINF1* variants. We believe that this case illustrates the role of research in identifying new genes associated with bone fragility.

## CASE REPORT

This case report was approved by the institutional review board of the University Hospital of the Ribeirao Preto Medical School at USP (CAAE: 38807320.5.0000.5440). The patient provided written informed consent.

**Figure 1:**
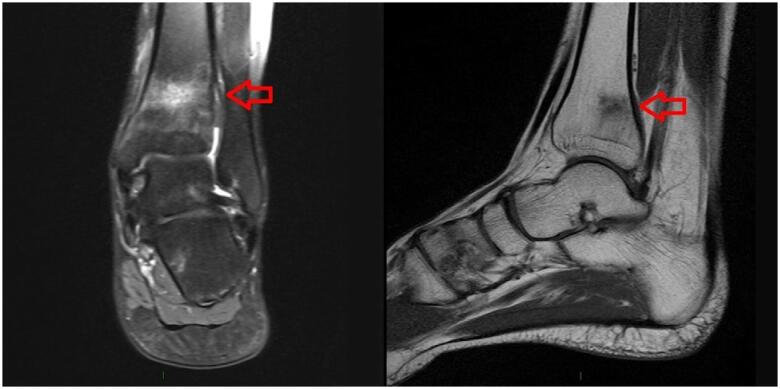
Magnetic resonance imaging showing coronal T2 and sagittal T1 views of the patient's fracture in the left distal tibia.

A 33-year-old woman attended our outpatient clinic with a history of four previous fractures that had occurred in mild or moderate mechanical stress situations after she started regularly attending a gym. Her first fracture occurred 4 years before the medical consultation, in the left distal tibia, after a 20-minute run on a treadmill, and was confirmed by magnetic resonance imaging (MRI) ( Figure 1 ). She sustained two subsequent fractures in the same region during light runs, 3 and 7 months after the first fracture. The fourth fracture affected the right distal tibia and occurred while the patient practiced with a jump rope. No other fractures were described. She practiced running mostly indoors and after warming up. She had no history of eating disorders. She had mild dyslipidemia, which was treated with rosuvastatin 10 mg, but had no family history of dyslipidemia. She was taking vitamin D3 2000 IU/daily since the first fracture but had sufficient vitamin D levels. She was otherwise healthy and had no previous clinical conditions.

Her menses started at age 13 and were always regular. At the time of the first appointment, she was nulliparous and was not using contraceptive pills. She had no previous use of glucocorticoids. She denied smoking, had a low intake of alcohol, consumed a low amount of dairy products, and had reduced sun exposure. Her parents were unrelated. Her mother had a medical history of osteopenia and had sustained a forearm fracture in her youth; her father was healthy. Her brother was healthy but also had a history of fractures associated with the practice of radical sports.

The patient was 164.5 cm tall and weighed 67.1 kg. Her body mass index was 24.79 kg/m² at the time of the first appointment. Her sclera was slightly darkened, and she had no visible bone deformities or disorders of tooth development. No abnormality was detected in her clinical examination. She had no pathological features in her lower limbs, her legs were aligned, and her feet had a normal arch.

The patient's biochemical tests revealed levels of total serum calcium of 9.6 mg/dL (reference values [RV] 8.5-10.5 mg/dL), 25-hydroxyvitamin D (25(OH)D) of 30 ng/mL (RV 20-50 ng/mL), phosphorous 3.4 mg/dL (RV 2.5-5.0 mg/dL), alkaline phosphatase 60 U/L (RV 35-104 U/L), serum creatinine 0.7 mg/dL (RV 0.7-1.5 mg/dL), TSH 2.4 mIU/L (RV 0.4-4.5 mIU/L), PTH 31 pg/mL (RV 10-65 pg/mL), prolactin 6.5 μ/L (RV < 31 μ/L), LH 9.0 IU/L (RV < 12 IU/L), total cholesterol 260 mg/dL, LDL-cholesterol 174 mg/dL, triglycerides 298 mg/dL, and normal complete blood count.

The initial bone densitometry (Hologic Horizon, Waltham, MA, USA) report indicated: lumbar spine (L1-L4) 0.945 g/cm², Z-score −0.5; total hip 0.845 g/cm², Z-score −0.8; femoral neck 0.688 g/cm², Z-score −1.5. Her most recent densitometry, 5 years after the first, showed: L1-L4 0.976 g/cm², Z-score −0.6; total hip 0.806 g/cm², Z-score −1.0; femoral neck 0.652, Z-score −1.6.

Since the patient had recurrent fragility fractures, a next-generation sequencing (NGS) panel for bone fragility disease was performed at our Genomic Medicine Center through sequencing of the following 13 genes: *COL1A1, COL1A2, SERPINH1, FKBP10* , *SP7, BMP1, TMEM38B, WNT1* , *IFITM5, SERPINF1, CRTAP, LEPRE1* , and *PPIB* . DNA was extracted from blood samples from the patient, her mother, and her brother using the Super Quik-Gene-Rapid DNA Isolation kit (Promega Corp., Madison, WI, USA). Sequencing was performed using the Ion Personal Genome Machine (PGM; Life Technologies, Darmstadt, Germany) as per the manufacturer's instructions with the 200-bp single-end run configuration. The analyses of raw data mapping (against GRCh37/hg19), calling of variants, and variants annotation were processed using the Torrent Suite Software, version 4.4.2, the Torrent Variant Caller Plugin, and the Ion Reporter Software, version 5.16.0.2, respectively (all by Life Technologies).

**Figure 2:**
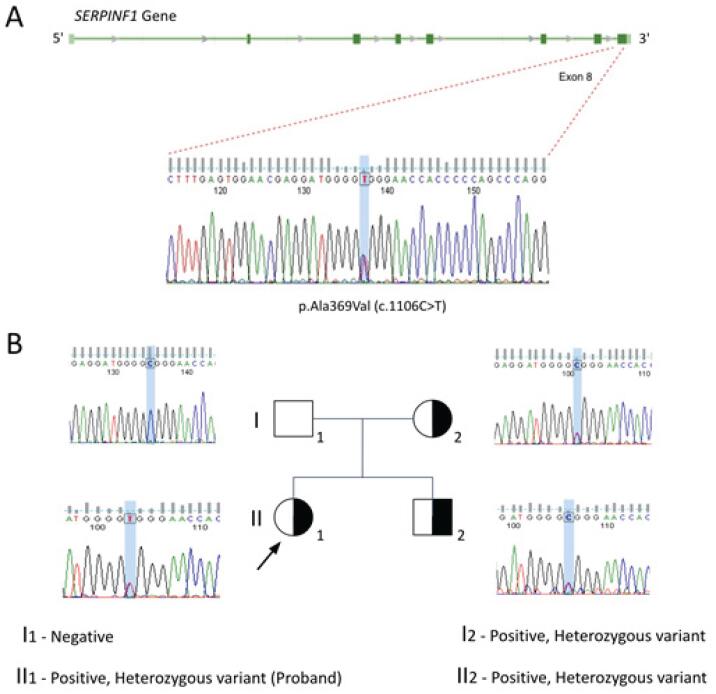
( **A** ) Heterozygous variant in the *SERPINF1* gene found in a female patient with multiple fractures. ( **B** ) Family genogram of the *SERPINF1* variant affecting three family members, including the patient, her mother, and a brother. The validation of the variant and the analysis of family segregation were performed after amplifying and sequencing the target region by the Sanger method following the manufacturer's recommendations (Applied Biosystems Genetic Analyzer, ThermoFisher Scientific, Waltham, MA, USA).

We identified a heterozygous variant – p.Ala369Val (c.1106C>T) rs368630571 – in the *SERPINF1* gene that had no description of clinical significance in the ClinVar platform but was likely associated with slight bone fragility. The same variant was identified in the other family members ( Figures 2a and 2b ). This is a missense variant, located in exon 8. It was defined as potentially pathogenic by the UMD-predictor (score 69) and CADD (score 16.17) software applications ( 5 , 6 ) and had a global allele frequency of 0.0001273%, according to the Genome Aggregation Database (gnomAD) ( 7 ). According to the recommendation criteria by the American College of Medical Genetics and Genomics and the Association for Molecular Pathology ( 8 ), this variant is classified as likely benign (PM2, PP3, PP4, BP1, and BP4). This classification was supported by the Genetic Variant Interpretation Tool ( 9 ) and VarSome ( 10 ) platforms. The classification of the variant followed the recommendations of the American College of Medical Genetics and Genomics and the Association for Molecular Pathology ( 8 ). The variant was not identified in the ABraOM database ( 11 ). *SERPINF1* variants are associated with type VI OI. Since this is an autosomal recessive disease, individuals with heterozygous variants are expected to be phenotypically normal.

## DISCUSSION

The most well-known forms of OI with autosomal dominant inheritance are the classical types I-IV, which are associated with variants in the *COL1A1* and *COL1A2* genes. More recently, variants in collagen-related genes have been described as causing other forms of OI. The *SERPINF1* is included in this newer group of autosomal recessive inheritance and expresses a collagen chaperoning protein. The homozygous variant in this gene causes OI independently from collagen type-I synthesis. *SERPINF1* encodes the pigment epithelium-derived factor (PEDF), which appears to have a high affinity to collagens of the extracellular matrix ( 12 ) and acts as an inhibitor of bone resorption ( 13 ). *SERPINF1* is described as a gene associated with rare monogenic diseases that have an impact on bone mass and strength ( 14 ), including in the Brazilian population ( 15 ). In homozygosity, bone fragility is well recognized, but in heterozygosity, there is still a gap in our knowledge regarding the occurrence of alterations. The study of candidate alleles for bone fragility is still growing, and the report of single individuals or families with variants can help fill this gap.

In a previous study, Al-Jallad and cols. (2014) described that heterozygous *SERPINF1* variant carriers had no detectable abnormalities in bone mass or adipose tissue distribution ( 4 ). Compared with non-carriers, carriers of this variant had similar body mass indexes and levels of bone turnover markers and no significant differences in bone mineral density, with the only exception for levels of PEDF, which were significantly lower in the carrier group. Although this protein has several potential functions, its deficiency apparently expresses as a specific skeletal phenotype with no other clinical manifestations.

Our patient also had a normal clinical phenotype, no biochemical abnormalities, and bone mineral density that was adequate for her age. However, she had a clinically significant outcome of four fractures in circumstances that combined stress and fatigue fracture. Measurement of PEDF levels was not done in the present case. Al-Jallad and cols. (2014) mentioned that the clinical effect of variants in only one *SERPINF1* gene (in heterozygosis) is lower than the one observed in genes *WNT1* and *LRP5* , which in heterozygosis lead to osteoporosis, although there was no information from the investigated individuals on activities that required physical effort ( 4 ). It is important to point out that *LRP5* and *WNT1* play a clear role in the *WNT* signaling pathway, while the *PEDF* mechanisms on bone fragility remain to be determined.

One concern raised during the investigation of this patient was the differential diagnosis with stress fractures in female athletes, since women are at increased risk for this type of fracture ( 16 ). The female athlete triad comprises caloric restriction, amenorrhea, and low bone mass. None of these features were part of the clinical profile of our patient. Moreover, our patient did not meet the criteria for this condition since her pain onset was acute and not insidious, as typically occurring in stress fractures ( 17 ). She had no risk factors for low bone density – such as smoking, high alcohol intake, rheumatoid arthritis, or glucocorticoid use – or a low Z-score on bone densitometry. Despite her low consumption of dairy products, this scenario is compatible with the one found in the Brazilian population. According to the International Osteoporosis Foundation (IOF), the mean daily calcium intake in the Brazilian population is 505 mg; thus, we do not attribute her history of fractures to calcium intake alone. Altogether, the clinical presentation of our patient seems to blend the two types of stress fracture, meaning that the fragility fracture occurred with mechanical stress insufficient to provoke a fatigue fracture in a young woman. These circumstances signaled an underlying cause for the recurrent fractures. Even though the variant is classified as likely benign, we hypothesize that we are facing a case of haploinsufficiency, which occurs when the normal phenotype requires the protein product of both alleles, and a 50% reduction in protein production results in an abnormal phenotype ( 18 , 19 ).

Using genomic, functional, and evolutionary characteristics, a group of researchers put together a predictive model of haploinsufficient genes and compiled a list of 12,443 genes ( 20 ). Interestingly, the *SERPINF1* gene is one of the predicted genes on the list. Its haploinsufficient profile still needs to be experimentally validated. However, depending on the clinical profile, individuals who are heterozygous for the *SERPINF1* gene may still exhibit subtle abnormalities, which should be kept in mind when patients are evaluated.

In conclusion, underneath repeated fragility fractures in young individuals usually lies a predisposing secondary condition, which may not be clinically or biochemically detectable. The findings of the present study show, for the first time in the literature, that a “stress fracture” can be a clinical manifestation in a patient harboring a heterozygote variant in *SERPINF1* . The present study highlights the relevance of molecular investigation in special cases of osteoporosis, a neglected disorder, even when repeated fractures are part of the clinical scenario. A larger number of studies and consistent investigations are nonetheless needed to elucidate the bone phenotype in carriers of *SERPINF1* variants and their association with bone fragility. When possible, a thorough investigation is recommended since it can further contribute to the identification of candidate genes for bone fragility.
